# A spontaneous nonhuman primate model of inherited retinal degeneration

**DOI:** 10.1172/jci.insight.190807

**Published:** 2025-05-06

**Authors:** Wei Yi, Mingming Xu, Ying Xue, Yingxue Cao, Ziqi Yang, Lingli Zhou, Yang Zhou, Le Shi, Xiaomei Mai, Zehui Sun, Wenjie Qing, Yuying Li, Aolun Qing, Kaiwen Zhang, Lechun Ou, Shoudeng Chen, Elia J. Duh, Xialin Liu

**Affiliations:** 1 State Key Laboratory of Ophthalmology, Zhongshan Ophthalmic Center, Sun Yat-sen University, Guangdong Provincial Key Laboratory of Ophthalmology and Visual Science, Guangzhou, China.; 2 Department of Ophthalmology, Affiliated Hospital of Nantong University, Nantong, China.; 3 Department of Ophthalmology, Johns Hopkins School of Medicine, Baltimore, Maryland, USA.; 4 Central Laboratory, the Fifth Affiliated Hospital, Sun Yat-sen University, Zhuhai, Guangdong, China.

**Keywords:** Genetics, Ophthalmology, Genetic diseases, Mitochondria, Retinopathy

## Abstract

Inherited retinal degenerations (IRDs) are important causes of progressive, irreversible blindness. Hereditary macular diseases, in particular, are significant in their effect on the specialized, central cone photoreceptor–rich macula responsible for high resolution vision. Autosomal dominant Best vitelliform macular dystrophy (BVMD), caused by variants in the *BEST1* gene, is one of the most common inherited macular dystrophies. Gene therapies have emerged as promising treatments for IRDs, but a lack of suitable animal models has hindered progress both in treatments and in understanding the mechanisms underlying macular diseases. Here, we report a *Macaca fascicularis* carrying a heterozygous potential pathogenic *BEST1p.Q327E* variant that disrupts the BEST1 ion channel by destabilizing the A195 helix, mirroring the structural perturbations seen in certain human pathological mutants. Longitudinal imaging over 2 years revealed progressive macular changes, including subfoveal cleft enlargement, lipid-rich deposit accumulation, retinal pigment epithelium (RPE) disruption, and central-to-peripheral photoreceptor degeneration, recapitulating early human BVMD pathology. Histopathology demonstrated diminished BEST1 expression, attenuation of the RPE-photoreceptor interface, and 2 distinct types of lipid deposits, including heretofore unappreciated cone mitochondrial-enriched lesions, highlighting selective cone mitochondria vulnerability. This is, to our knowledge, the first nonhuman primate model of inherited macular dystrophy, and it links *BEST1* mutations, mitochondrial dysfunction, and progressive macular degeneration, offering new insights into BVMD pathophysiology and highlighting its utility for studying disease progression and potential therapeutic interventions.

## Abstract 2

This study presents the first non-human primate model of an inherited macular disease, specifically Best disease, promoting treatment efforts and providing novel pathophysiological insights

## Introduction

Inherited retinal degenerations are an important cause of progressive, irreversible blindness. Hereditary macular diseases, in particular, are significant, since they affect the specialized, central cone photoreceptor-rich macula that is responsible for high resolution vision. The retinal pigment epithelium (RPE) is critical in the health of the photoreceptors, and, indeed, disorders of the RPE affect more than 10 million individuals in the U.S. alone, from inherited retinal degenerations and age-related macular degeneration (1). Notably, the first FDA-approved gene therapy for an inherited disease, Luxturna, delivers a normal copy of the *RPE65* gene into RPE cells, based on a pivotal Phase III trial (2).

Autosomal dominant Best vitelliform macular dystrophy (BVMD, also called Best disease), caused by variants in the *BEST1* gene, is one of the most common inherited macular dystrophies (3–5). The typical clinical phenotype of BVMD is a bilateral egg-yolk appearance in the central macula, characterized by subfoveal cleft formation and subretinal accumulated hyperreflective material, as observed via Spectral-domain optical coherence tomography (SD-OCT) imaging (6). BVMD progresses through five stages based on fundus features, with yolk lesions that can be reversible or recurrent, although progression is rarely predictable across different patients (7). Genetic evidence of pathogenic variants in the *BEST1* gene is now considered as the gold standard for diagnosing this disease (3, 4). *BEST1* encodes Bestrophin-1, a Ca^2+^-activated Cl^–^ channel in the basolateral membrane of the RPE (8, 9). Many pathogenic *BEST1* variants significantly decrease chloride ion conductance in cellular models, disrupting intra- and extracellular ionic equilibria of the RPE (10, 11). This disruption damages the RPE-photoreceptor interface, affecting the phagocytosis and uptake of photoreceptor outer segments (12). Although *BEST1* is pan-expressed in the RPE throughout both the macular and peripheral retina, the mechanisms by which pathogenic *BEST1* variants selectively cause macular lesions in BVMD remain poorly investigated. In addition, although BVMD is a disease condition ideally suited to gene- and cell-based therapies (9), the lack of an animal model that recapitulates the unique macular phenotypic changes has been a major limiting factor.

Nonhuman primates (NHP) are the most informative model for retinal diseases due to their anatomic, physiologic, and genetic similarities to human (13, 14). NHP models are particularly critical for diseases of the macula, the specialized cone photoreceptor-rich structure absent in all other animal models. This structure, including the central fovea, is responsible for the high-resolution central vision in humans and NHP and often undergoes unique pathophysiologic changes that do not occur elsewhere in the retina (14). A major challenge is the lack of such models due to the tremendous resources required for their creation or identification, especially as both phenotypic and genotypic confirmation are needed (14). Strikingly, the only primary retinal disease model for which genotypic and phenotypic confirmation are known is a naturally occurring NHP model for achromatopsia, caused by a homozygous R565Q missense mutation in the catalytic domain of *PDE6C* (15). This disease causes a loss of cone function throughout the entire retina. To date, there remain no available NHP models of inherited macular conditions.

In this study, we report the identification of a *Macaca fascicularis* carrying a heterozygous deleterious *BEST1 p.Q327E* variant, exhibiting characteristic clinical and histopathological features resembling earlier stages of BVMD. We also demonstrate new, previously unreported insights into cellular changes, particularly damaged cone mitochondria, that only occur in the macular region. Our study establishes the first NHP model of inherited macular dystrophy and highlight the importance of NHP models in enabling insights into the unique pathophysiological processes in macular diseases, including dysfunction in RPE-photoreceptor interactions, cone mitochondrial damage, and downstream sequelae.

## Results

### Identification of a macaque with a heterozygous deleterious BEST1p.Q327E variant.

**Figure 1:**
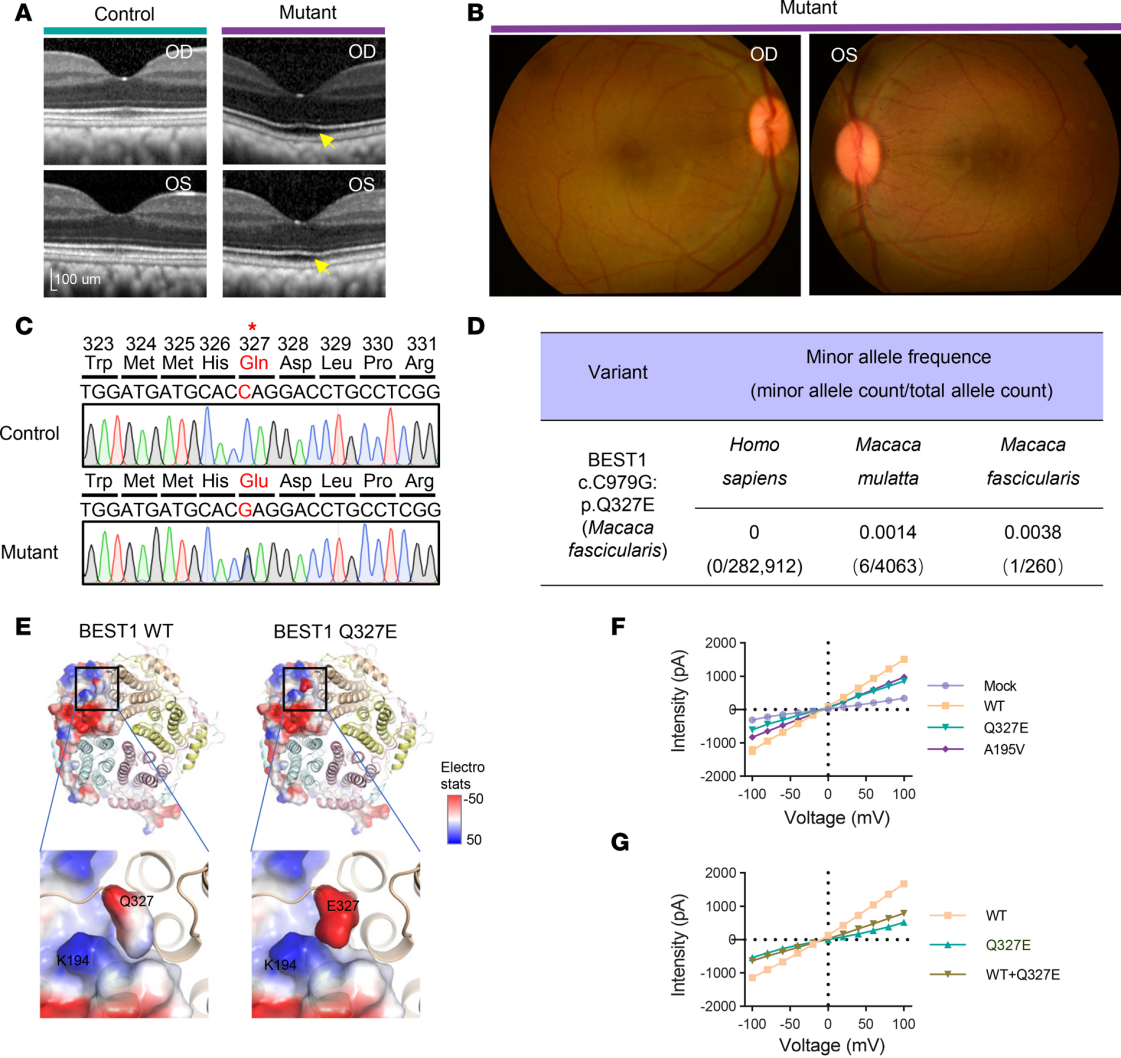
Identification of a macaque with a heterozygous deleterious *BEST1p.Q327E* variant. (**A**) SD-OCT imaging reveals a small subfoveal cleft (as indicated by the yellow arrow) in the abnormal animal (6.8 years old), compared with the age-matched normal macaque (6.8 years old). (**B**) Fundus images from the mutant macaque did not show significant pathologic changes, including vitelliform lesion. (**C**) Sanger sequencing showed that the abnormal *Macaca fascicularis* carries a heterozygous *BEST1p.Q327E* variant. (**D**) The allele frequency of the *BEST1:c.C979G: p.Q327E* variant in *Homo sapiens*, *Macaca mulatta,* and *Macaca fascicularis*. (**E**) Structural modeling of the human Bestrophin-1 (hBest1) pentamer structure (PDB: 8D1K) showed that substitution of the glutamine (Q) side chain to glutamic acid (E) increases negative charge and enhances its electrostatic interactions with the main chain of K194 in the adjacent protomer. Q327/E327 in protomer 1 and adjacent protomer 5 are showcased with a protein electrostatic potential rendering, ranging from red (–50) to blue (50). (**F**) Current-voltage relationship of HEK293T cells transiently expressing WT or BEST1 variants, measured by whole-cell patch clamping for calcium-activated chloride ion conductance. The Q327E and A195V exhibited smaller currents compared with the WT. The mock group transfected with empty vectors showed the lowest current signal. Data are presented as mean ± SEM. *n* = 6–9. *P* < 0.001 for both Q327E and A195V, compared with WT mfBest1. *P* values were calculated using 2-way ANOVA. (**G**) Cotransfection of Q327E and WT BEST1 plasmids in a 1:1 ratio significantly reduced the current from WT BEST1. Data are presented as mean ± SEM. *n* = 6–10. *P* < 0.001 for Q327E+WT compared with WT mfBest1. *P* values were calculated using 2-way ANOVA.

In a previous population screening of a *Macaca fascicularis* (crab-eating macaque) cohort from South China that included fundus photography and SD-OCT examination (16), we identified a 6.8-year-old male (approximately in late adolescence) exhibiting macular abnormalities. SD-OCT imaging revealed an enlarged subfoveal cleft and reduced reflectivity in the foveal outer retinal layers (Figure 1A), despite otherwise normal findings on color fundus photography (Figure 1B).

We conjectured that this young individual might represent an early-stage case of inherited macular dystrophy. We therefore conducted whole-genome sequencing of the abnormal animal, along with 29 randomly selected individuals from the macaque cohort. Our analysis focused on the single nucleotide variants (SNVs) in genes associated with inherited macular dystrophies, including *BEST1*, *ABCA4*, *EFEMP1*, *ELOVL4*, *IMPG1*, *PROML1*, *PRPH2*, *RS1,* and *TIMP3* (17, 18). Notably, a heterozygous *BEST1p.Q327E* variant was exclusively detected in the abnormal animal. Sanger sequencing further validated the presence of this variant (Figure 1C). This mutation substitutes glutamine (Q) with glutamic acid (E) at residue 327. The high conservation of the Q327 residue across vertebrates underscores its critical role in BEST1 function (Supplemental Figure 1A; supplemental material available online with this article; https://doi.org/10.1172/jci.insight.190807DS1).

The *BEST1p.Q327E* variant has not been previously reported in human patient databases such as Clinvar and The Human Gene Mutation Database (HGMD). To determine its potential pathogenicity, we examined allele frequency (AF) in the population. According to American College of Medical Genetics and Genomics (ACMG) guidelines for interpreting sequence variants in inherited Mendelian diseases, an AF < 0.05 is considered a critical threshold for identifying potentially pathogenic variants. A recent study profiling the pathogenicity of SNVs in rhesus macaque cohorts suggests an AF of 0.038 in the rhesus macaque population (mCED database) and 5.1 × 10^−3^ in the human population (gnomAD database) as stringent cutoffs (19). The AF of *BEST1p.Q327E* is 0 in gnomAD and 0.00137 in mCED (Figure 1D), both well below the established cutoffs. Additional Sanger sequencing of 100 randomly selected crab-eating macaques from the population revealed no other individuals carrying the *BEST1p.Q327E* variant, resulting in an AF of 0.0038 (Figure 1D). These findings support the classification of *BEST1p.Q327E* as a rare, putative pathogenic variant.

To explore the potential functional consequence of Q327E, we performed in silico structural modeling based on the crystallographic structure of human BEST1 (20). The substitution of the glutamine side chain to glutamic acid increases a negative charge, enhancing its electrostatic interactions with the backbone of K194 in the adjacent protomer (Figure 1E). This alteration could potentially cause the helix spanning residues 194–195 to shift away from the ion trafficking center, which affect the aperture conformation of the BEST1 ion channel. We investigated whether known human Best mutations could induce similar disruptions in the stability of the A195 helix. Interestingly, our structural modeling revealed that several previously reported pathogenic mutants of human Best disease, specifically A195V, M325H, and I201T (21–23), could have a similar impact (Supplemental Figure 1, B–E).

In silico predictions suggested that the Q327E variant could impair channel function. To validate this hypothesis, we conducted whole-cell patch to assess calcium-activated chloride ion conductance in HEK293T cells transiently expressing WT or BEST1 variants, including Q327E and A195V. Compared with the robust chloride ion conductance observed with WT BEST1, both the Q327E and A195V nearly abolished chloride ion currents (Figure 1F). Cotransfection of Q327E and WT BEST1 plasmids in a 1:1 ratio significantly reduced the current from WT BEST1 (Figure 1G), confirming that the heterozygous BEST1 Q327E mutation is sufficient to impair overall BEST1 channel function, consistent with the dominant inheritance pattern. These findings demonstrate that this animal carries a deleterious *BEST1p.Q327E* variant.

### Progressive clinical phenotypes of stage 1 BVMD in the mutant macaque.

Human *BEST1* mutations are associated with diverse clinical presentations of BVMD. In the previtelliform stage (Stage 1), patients often present with a normal fundus or subtle alterations in the RPE. Patients may progress to Stage 2 with characteristic vitelliform deposits at different ages, from early adolescence to over 50 years of age (6, 24, 25). Recent advances in high-resolution SD-OCT imaging have identified early-stage changes, including subretinal material accumulation, alterations of the RPE and inner/outer segment (IS/OS) junctions, and macular retinal thickness, even in the absence of overt vitelliform lesions (25–28).

**Figure 2:**
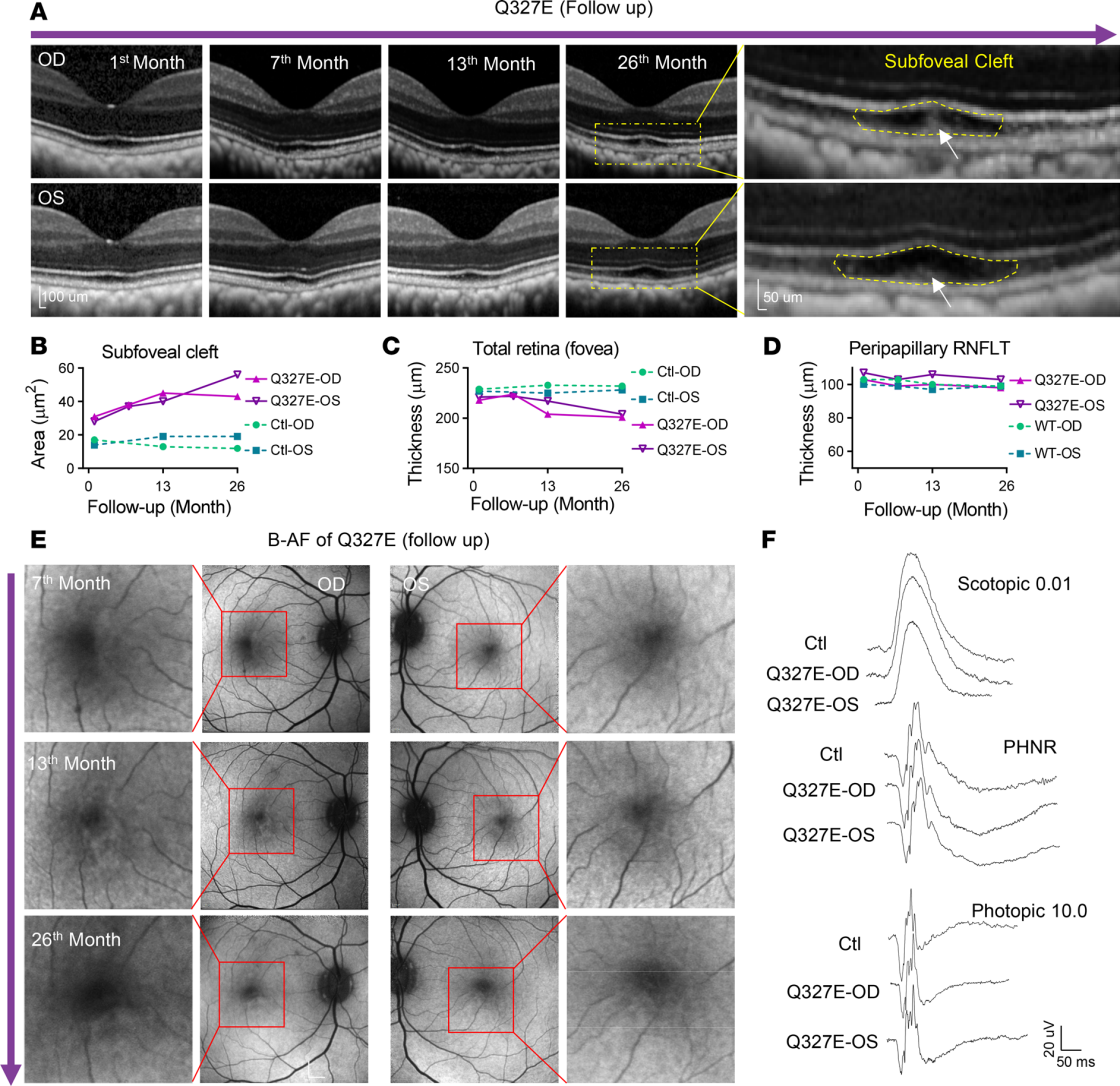
The Q327-mutant macaque exhibited clinical manifestations of Stage 1 Best vitelliform macular dystrophy. (**A**) SD-OCT scans of the mutant macaque and an age-matched control over 26 months of follow up. The mutant animal exhibited a progressively enlarging subfoveal cleft (area outlined in yellow) in both eyes. Deposits in the subfoveal cleft were also observed (white arrows). The first month SD-OCT image in **A** is the same as the mutant SD-OCT image shown in Figure 1A. (**B**) The subfoveal cleft areas from both eyes of the mutant macaque were enlarged during the 26 months of follow up. (**C**) Total retinal thickness of the mutant macaque decreased over time in both eyes, while that of the control remained unaltered. (**D**) The thickness of the parapapillary retinal nerve fiber layer remained unchanged in both the mutant and age-matched control macaque. (**E**) Blue light fundus autofluorescence (B-AF) examinations disclosed a slight increase in the size of the autofluorescence lesion in the right eye over 19 months from the seventh-month follow-up visit to the 26th-month follow-up visit. The autofluorescence lesion in the left eye was not as prominent.The red box indicates the enlarged area in the adjacent image. (**F**) Representative ERG waveforms of mutant animal and control, including scotopic 0.01, photopic 10.0, and the photopic negative response (PhNR). ONL, outer nuclear layer; B-AF, blue light fundus autofluorescence; ERG, electrophysiology.

We next conducted longitudinal SD-OCT examination of the Q327E mutant macaque over two years, revealing progressive structural changes consistent with early BVMD. Subfoveal cleft enlargement and accumulation of subretinal deposits were observed (Figure 2A). The cleft area increased during the first year and further expanded during the second year in the right eye (Figure 2B). Foveal retinal thickness decreased in both eyes over two years (Figure 2C), consistent with cone degeneration. In contrast, peripapillary retinal nerve fiber layer thickness (RNFLT) remained unaffected throughout the follow-up period (Figure 2D and Supplemental Figure 2A), consistent with preservation of inner retinal cell integrity.

Blue light fundus autofluorescence (B-AF) imaging showed irregular hyperfluorescence in the macula of the right eye, consistent with RPE changes (Figure 2E). Fundus imaging remained normal without classic vitelliform deposits at the last visit at age 8.9 years (Supplemental Figure 2B). Similarly, full-field electroretinography (ffERG) responses, including scotopic, photopic, and photopic negative responses (PhNR), were comparable between mutant and control animals (Figure 2F), consistent with early-stage BVMD phenotypes in human patients (29).

In summary, the Q327E-mutant macaque exhibited progressive clinical phenotypes highly resembling the early-stage BVMD observed in human patients, which may recapitulate a pathological progression from the previtelliform stage to early vitelliform stage.

### Central-to-peripheral pattern of macular degeneration.

**Figure 3:**
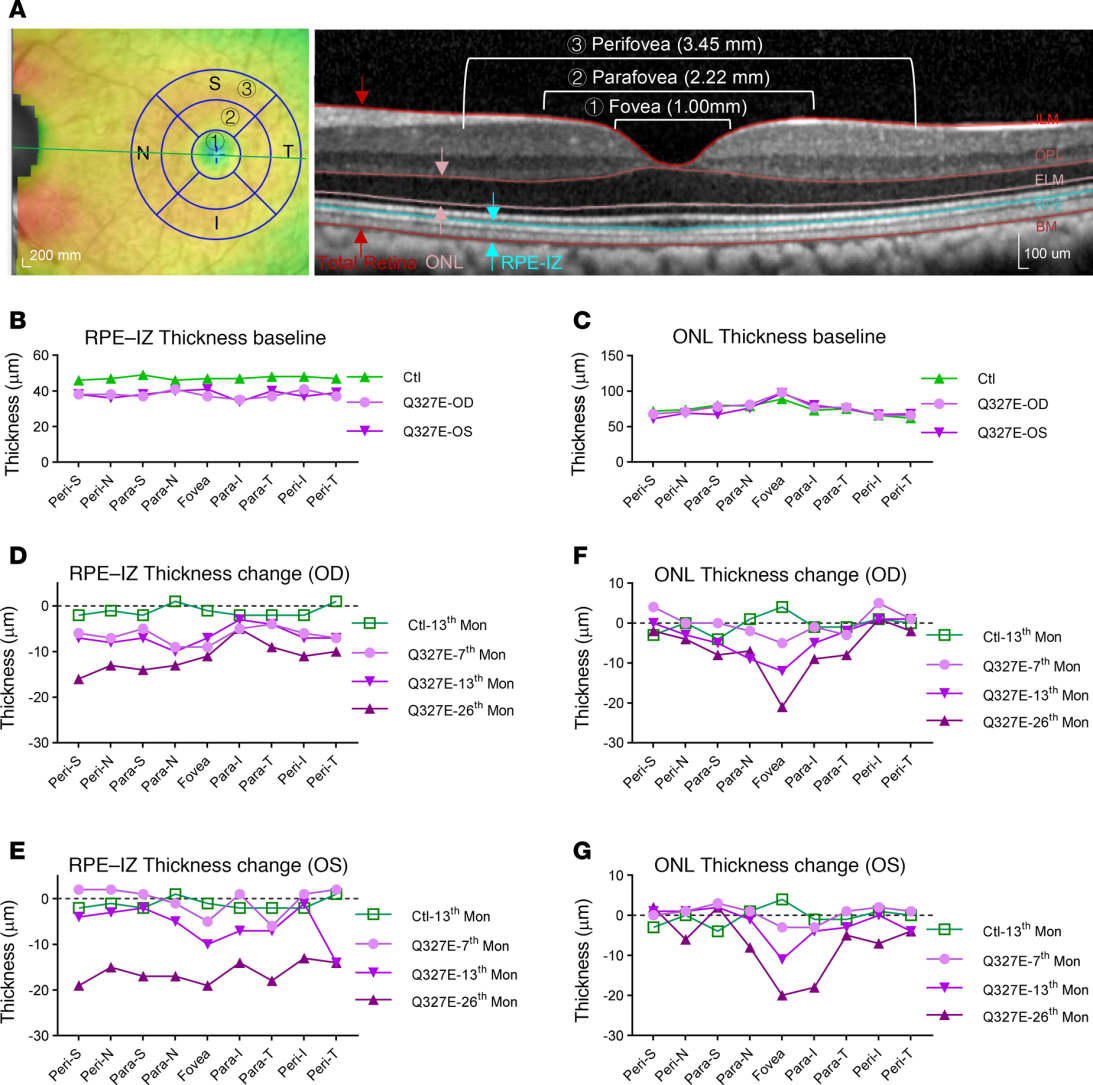
Change in outer nuclear layer and RPE thickness over time in the Q327-mutant macaque. (**A**) Schematic diagram showing the four directions and anatomical subdivisions of the retina (left panel) and associated segmentation (right panel) for analysis. The retina was divided into nasal (N), inferior (I), temporal (T), and superior (S), relative to the fovea (no. 1), parafoveal region (no. 2), and perifoveal region (no. 3). The OCT boundaries used to measure total retinal thickness, thickness of the ONL, and thickness of the RPE-IZ region, respectively, are denoted (right panel). In this study, the fovea was defined as a 1.0 mm diameter ring centered on the foveola, the parafovea was defined as an annulus centered on the fovea between circles with diameters of 1.0 mm and 2.22 mm, and the perifovea was defined as an annulus centered on the fovea between the circles with diameters of 2.22 mm and 3.45 mm. The position of the foveola was determined by manually determining the central fovea on the horizontal scans. (**B** and **C**) The baseline thickness of RPE-IZ (**B**) and ONL (**C**) in the first OCT examination from the mutant macaque and the age-matched control macaque. (**D**–**G**) The thickness change of RPE-IZ and ONL during the follow-up visits compared to the initial examination are displayed. RPE-IZ, the RPE-Bruch’s membrane (BM) complex and the RPE-photoreceptor interdigitation zone; ONL, the outer nuclear layer.

Given the known pan-RPE expression of *BEST1*, we investigated the spatial progression of RPE and photoreceptor disruption in the Q327E mutant macaque using OCT-based thickness measurements in foveal (1 mm), parafoveal (2.22 mm), and perifoveal (3.45 mm) regions of the macula (Figure 3A). The thickness of outer nuclear layer (ONL), which contains the cell bodies of photoreceptors, and the thickness of the hyperreflective RPE-IZ layer, which includes the RPE-Bruch’s membrane (BM) complex and the RPE-photoreceptor interdigitation zone (30) were measured, respectively (Figure 3A).

At the initial visit, thickness of the RPE-IZ was slightly reduced across the macula in both eyes, with the foveal center of the right eye most affected (Figure 3B), aligning with the B-AF observations (Figure 2E). ONL thickness across the macula, however, remained comparable to the age-matched control, suggesting photoreceptors were preserved at the initial visit (Figure 3C).

Over the two-year observation period, RPE-IZ thinning progressively advanced across the macula. In the right eye, RPE-IZ thickness declined rapidly during the first year, followed by a slower rate of thinning in the second year (Figure 3D). In the left eye, RPE thinning occurred more gradually in the first year but accelerated significantly in the second year (Figure 3E). By the end of the observation period, RPE thinning was evident throughout the macula in both eyes.

Although RPE-IZ thinning occurred broadly across the macula, ONL thinning followed a distinct central-to-peripheral gradient in both eyes, with the macular center being the most pronounced (Figure 3, F and G). In the right eye, ONL thickness declined more rapidly during the first year compared with the second year, mirroring the time course of RPE-IZ thinning and subfoveal cleft enlargement (Figure 3F). In contrast, the left eye exhibited a slower ONL thinning pattern during the first year, with an accelerated decline at the macular center during the second year (Figure 3G). These observations suggest that ONL thinning, indicative of photoreceptor degeneration, was secondary to RPE-IZ disruption in this mutant macaque and was most pronounced in the macular center. The increased vulnerability of photoreceptors in the central macula to progressive degeneration during the previtelliform stage likely contributes to the development of the characteristic vitelliform lesion observed in the foveal region of BVMD patients during the vitelliform stage.

### Histopathological analysis reveals characteristic pathogenic features of BVMD.

**Figure 4:**
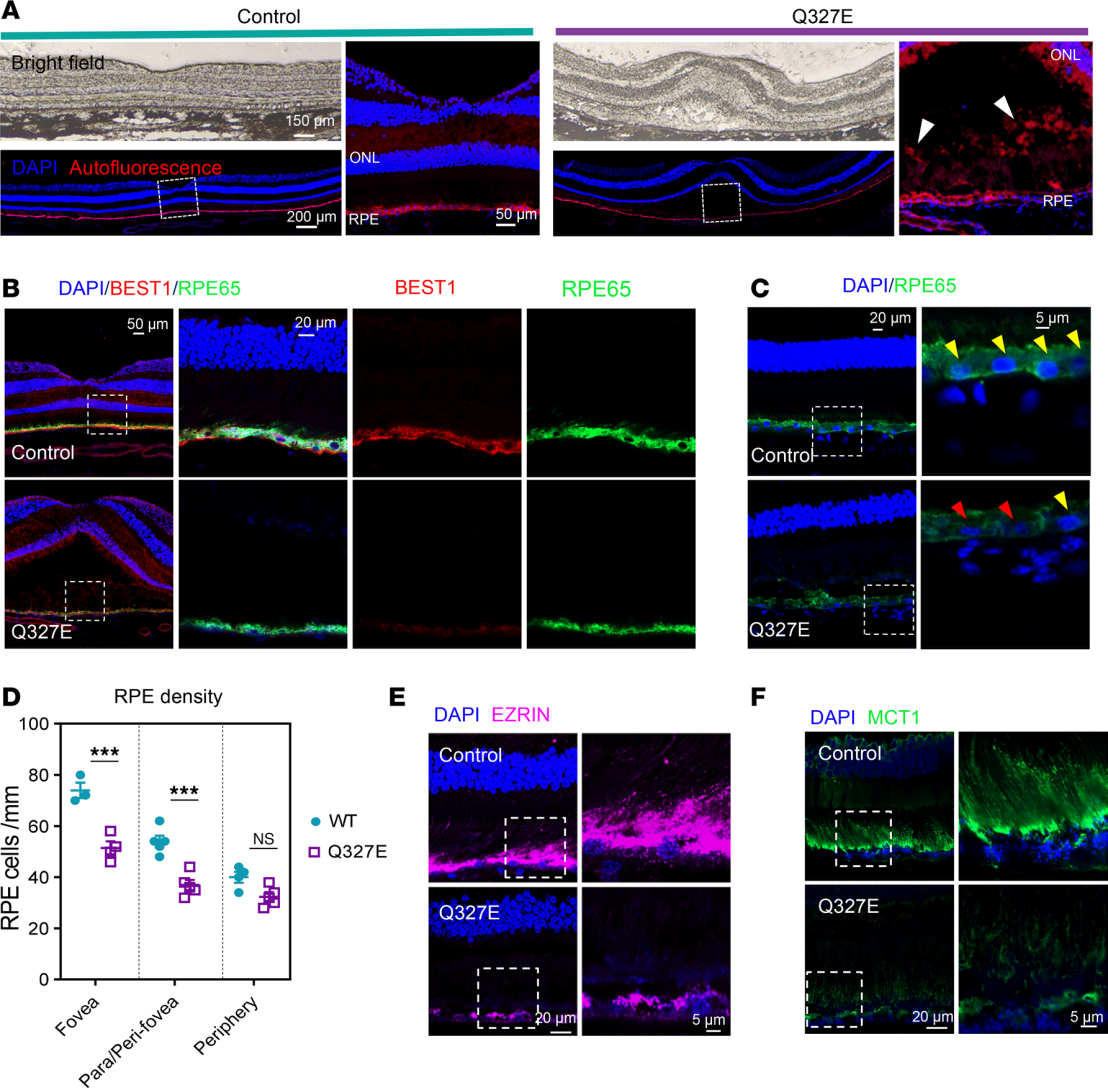
Histopathologic analysis reveals typical pathogenic features of BVMD. (**A**) Bright field and immunostaining imaging showing neuroretina detachment from the RPE in the mutant fovea, forming a cleft with the accumulation of autofluorescent deposits within it. Autofluorescence was detected at 555 nm (red), and nuclei were stained with DAPI (blue). (**B**) Immunostaining for BEST1 protein (red) and RPE cell marker RPE65 (green), with nuclei stained with DAPI (blue). (**C**) DAPI staining highlights fragmented RPE nuclei in the mutant animal. Fragmented nuclei are indicated by red arrowheads, while normal nuclei are indicated by yellow arrowheads. Costaining with RPE65 was performed to identify the nuclei of RPE cells. (**D**) The density of RPE cells was significantly reduced in the fovea and parafoveal/perifoveal regions and unchanged in the periphery in the mutant macaque. Each data point represents one fluorescent image. (**E**) Immunostaining for EZRIN (pink) indicated the absence of RPE apical microvilli in the macular region of the mutant retina. (**F**) Immunostaining for MCT1 (green) showed decreased signals in the apical surface and processes of the RPE and photoreceptor outer segments in the mutant macaque macula. Statistical significance was determined by 2-way ANOVA and Šidák’s post hoc comparisons. (***P < 0.001).

Histopathological analysis performed postmortem after the unexpected death of the macaque revealed hallmark pathogenic features consistent with clinical observations from OCT imaging. The mutant animal exhibited a prominent subfoveal cleft between the RPE and ONL, in contrast with an age-matched control. Autofluorescent deposits were also noted within the cleft (Figure 4A).

To assess BEST1 expression in the RPE, we performed immunostaining of retinal sections. In the control animal, BEST1 protein was predominantly localized in the basolateral plasma membrane of RPEs (Figure 4B). However, in the Q327E-mutant macaque, BEST1 signal was significantly reduced in the fovea (Figure 4B). Decreased BEST1 signal was also noted in the parafoveal and peripheral regions of the retina (Supplemental Figure 3, A and B), suggesting an overall decrease in BEST1 levels in the mutant animal.

As our in silico mutation analysis revealed no direct steric clash caused by Q327E, we next investigated whether Q327E affects BEST1 protein stability. In 293T cells with transient expression of WT BEST1 and BEST1-Q327E, no significant difference in protein levels was observed (Supplemental Figure 3C). In ARPE-19 cells with stable lentiviral expression, the Q327E mutant exhibited significantly lower BEST1 protein levels compared with cells expressing WT BEST1 (Supplemental Figure 3D). This reduction under long-term physiological conditions may be attributed to altered mobility and stability of the A195 helix, a structural component required for BEST1 pentamer assembly. Disruptions in this region could promote protein misfolding, decreased stability, and increased degradation, leading to progressive decline in BEST1 levels over time. These in vitro findings align with pathological observations in the mutant animal, supporting that Q327E induces protein instability.

Consistent with the OCT imaging measurements, the ONL was thinner in the of the mutant animal (Figure 4, B and C). Some RPE nuclei exhibited signs of disintegration and rupture (Figure 4C). Quantification of RPE density, as indicated by RPE nuclei, revealed a significant reduction in the mutant animal in the foveal, parafoveal, and perifoveal regions of the macula, indicating mild loss of RPE across the macula (Figure 4D).

Previous studies have shown that *BEST1* mutations disrupt the RPE-photoreceptor interface in rodent and canine models (12, 31). To further investigate this, we examined the structures of RPE apical projections using immunostaining for EZRIN and MCT1. EZRIN marks the apical microvilli of RPE, while MCT1 labels the apical processes of the RPE and photoreceptor outer segments (32, 33). In the control animal, strong EZRIN and MCT1 signal were observed, and the EZRIN-MCT1 labeled RPE-photoreceptor interface spanned 10–20 micrometers (Figure 4, E and F). However, in the macular regions of the mutant animal, both EZRIN and MCT1 signals were almost absent (Figure 4, E and F), suggesting retraction of RPE microvilli and disorganization of photoreceptor outer segment structures. The disruption of the EZRIN- or MCT1-labeled RPE-photoreceptor interface in the Q327E mutant animal could contribute to the 10–20 micrometer thinning of the RPE-IZ measured in the OCT imaging (Figure 3, D and E).

Collectively, these findings demonstrate that the mutant macaque exhibits characteristic pathological features of BVMD, including a prominent subfoveal cleft with hyperautofluorescent deposits, aberrant BEST1 expression, macular photoreceptor loss, disrupted RPE-photoreceptor interfaces, and RPE degeneration.

### Mitochondrial-containing lipid deposits contribute to the spatial selectivity of macular lesions in BVMD.

**Figure 5:**
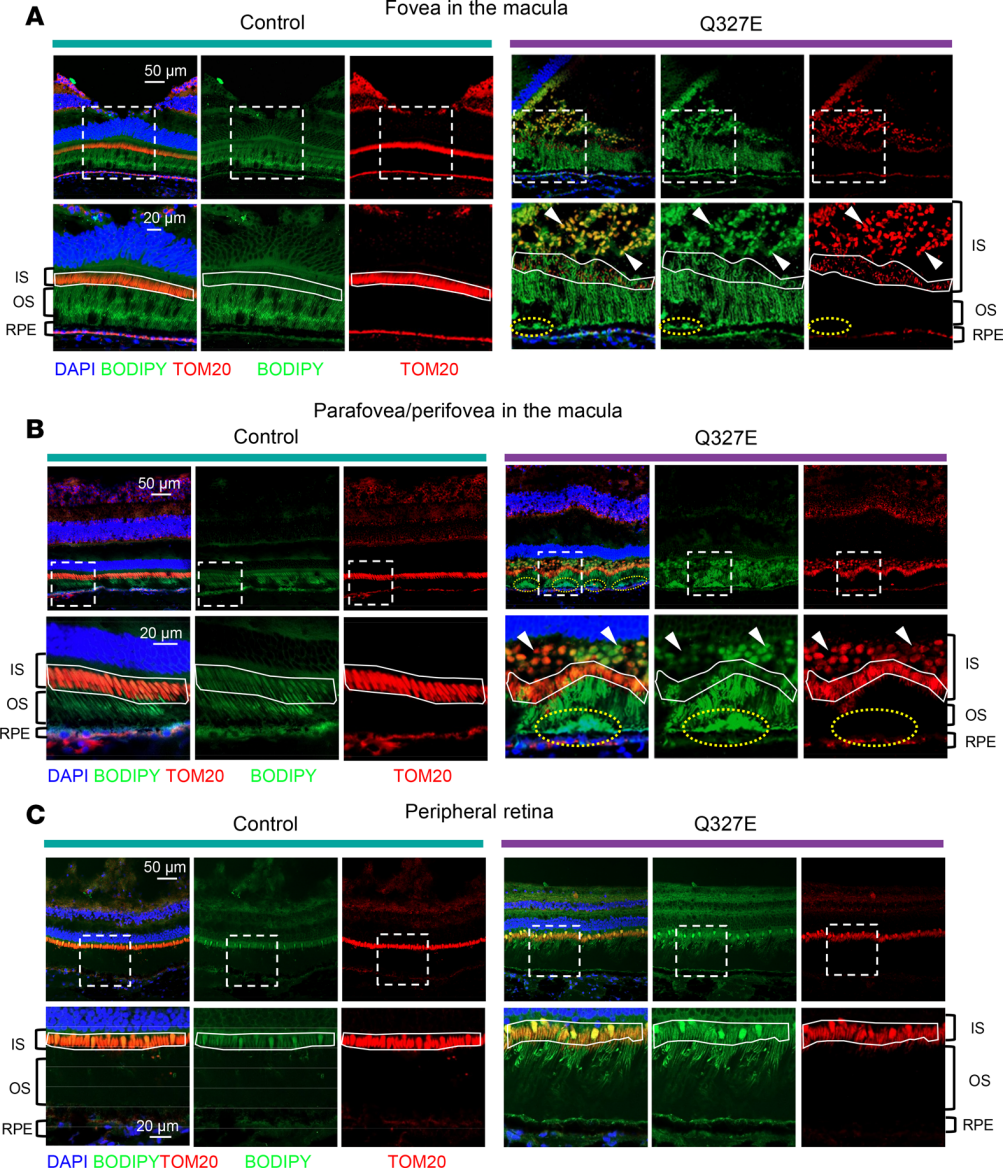
The Q327 mutant macaque exhibits mitochondrial-negative and -positive lipid deposits in the macula. (**A**–**C**) Representative images from immunostaining of BODIPY (green), TOM20 (red), and DAPI (blue) in different retinal regions, including fovea, parafovea/perifovea, and periphery. The bottom row shows zoomed-in views of the areas indicated by boxes in the top row. (**A** and **B**) In the fovea (**A**) and parafovea/perifovea (**B**), there was the accumulation of 2 types of BODIPY high deposits: deposits beneath the outer segments were TOM20 negative (indicated by yellow circles), while deposits above the outer segments were TOM20 positive (indicated by the white arrowheads). Mitochondria in the inner-segment region of photoreceptors, with low BODIPY signal, are outlined in white. A wavy distribution pattern of mitochondria was observed in the mutant animal. (**C**) The peripheral retina of the mutant animal showed a normally organized ellipsoid zone band with mitochondria, and deposits were rarely detected. TOM20, Translocase of outer mitochondrial membrane 20; EZ, ellipsoid zone; OS, outer segments; IS, inner segments.

Given that increased autofluorescence signals often indicate lipid deposits, we used neutral lipid dye BODIPY to examine the lipid compositions of these lesions. In the macular region of the mutant macaque, BODIPY staining revealed two distinct types of lipid-enriched deposits with high BODIPY signal, located either beneath or above the photoreceptor outer-segment region (Figure 5, A and B).

The first type of deposit, situated between the outer-segments and RPE cells, has been observed in *BEST1*-mutant canines and patients with BVMD (12, 34) and likely represents unphagocytosed photoreceptor membrane discs due to impaired RPE function (35, 36). The second type of deposit, found between the outer segments and the photoreceptor nuclear (Figure 5, A and B), has not been previously reported in BVMD and other maculopathies. H&E and oil red O staining further validated the presence and lipid-rich nature of these deposits (Supplemental Figure 4, A and B).

Given that the second type of deposits were in the region corresponding to the inner segments of the photoreceptors, which contain abundant mitochondria, we performed TOM20 immunostaining, a marker for the outer-mitochondrial membrane, to further characterize the nature of the deposit. The first type of deposit beneath the outer-segments was TOM20 negative, confirming it as outer-segment fragments. In contrast, the second type of deposit above the outer segments was TOM20 positive (Figure 5, A and B).

In the foveal center, where the photoreceptors are exclusively cones, these mitochondrial deposits were highly abundant, exhibiting a round, swollen morphology, suggesting that they were damaged (Figure 5A). Below these swollen mitochondrial deposits, we observed thin mitochondria with low BODIPY signal, resembling the morphology of cone mitochondria in the control fovea. However, these thin mitochondria were much less abundant in the mutant fovea compared with the control fovea (Figure 5A). These observations suggest that most cone mitochondria in the fovea were disrupted and the lipid-enriched second type of mitochondria deposits may represent accumulated damaged cone mitochondria.

In the parafoveal and perifoveal regions, where both cones and rods are present, the swollen mitochondria and BODIPY-high deposits were less prominent compared with the fovea center (Figure 5B). Wheat Germ Agglutinin (WGA) staining, which binds to glycoproteins on cell membranes, revealed that these deposits were enclosed within photoreceptor membranes, indicating that the damaged mitochondria remained intracellular (Supplemental Figure 4C). Beneath the round mitochondrial deposits in these regions, the remaining thin mitochondria with low BODIPY signal were arranged in a wave-like pattern, contrasting with the straight mitochondrial-enriched zone observed in the control retina (Figure 5B). This wave-like pattern may be due to the mechanical compression exerted by the two types of deposits.

In contrast with the macula, the periphery, where cones are sparse, showed normal outer segments and ellipsoid zone morphology, with barely any observed deposits (Figure 5C). These observations demonstrate a central-to-peripheral gradient of mitochondrial damage in photoreceptors, with the foveal center being the most severely affected. Considering the central-to-peripheral gradient of cone distribution and the exclusive presence of cones in the fovea, mitochondria in cones, rather than rods, appear to be especially vulnerable in BVMD. This selective vulnerability of cone mitochondria could contribute to the central-to-peripheral pattern of outer nuclear layer thinning observed in OCT measurements. Furthermore, the accumulation of damaged cone mitochondria and outer-segment fragments likely disrupts the microstructure of the outer retinal layers, which may ultimately lead to neurosensory retinal detachment from the RPE, contributing to the formation of the subretinal cleft.

### Alterations in outer retinal microstructure during disease progression.

**Figure 6:**
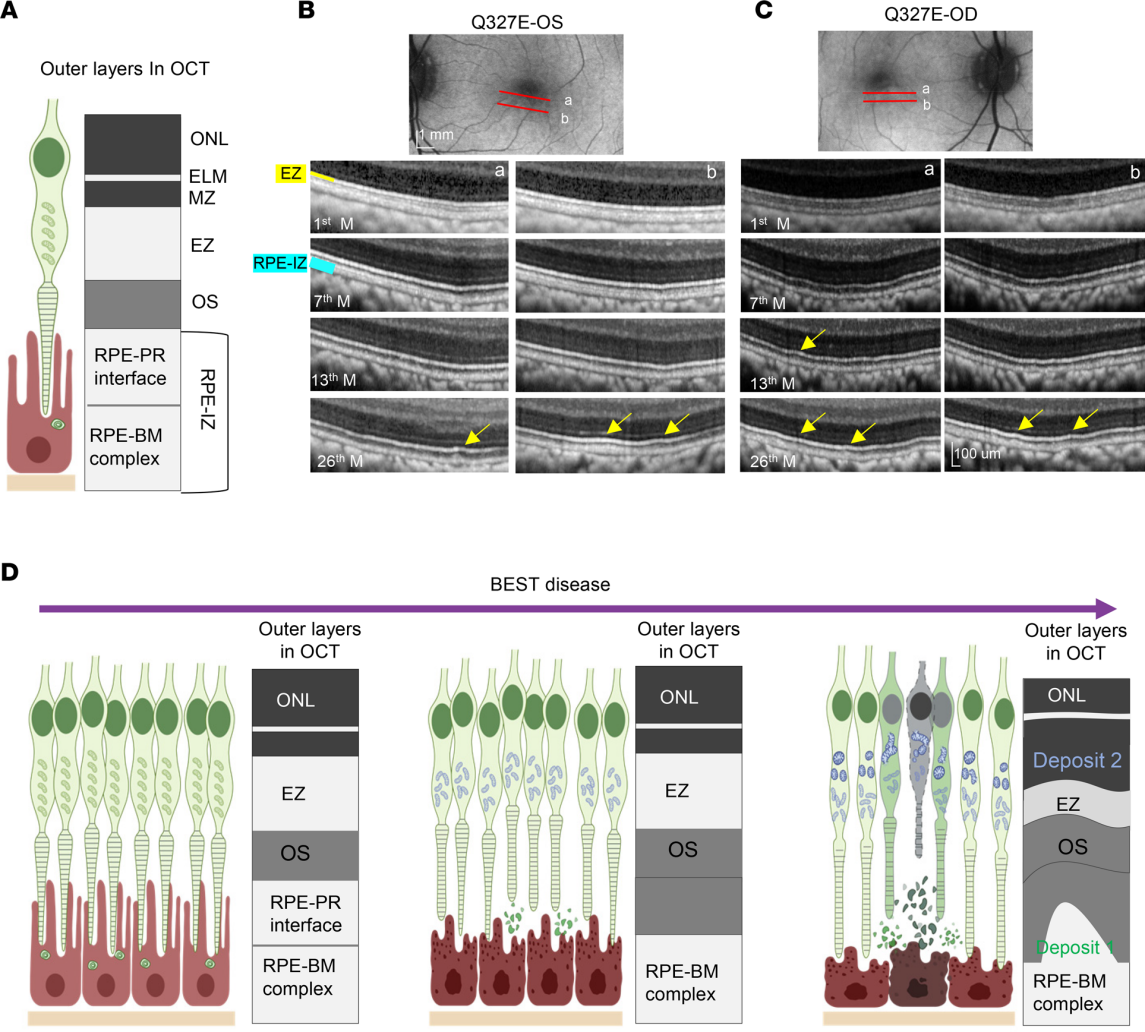
Alteration of outer retinal bands of OCT scans during disease progression in the Q327 mutant macaque. (**A**) Schematic representation of the structure of photoreceptor cells and RPE cells and their corresponding bands in the OCT image. ONL, outer nuclear layer; ELM, external limiting membrane; MZ, myoid zone of the photoreceptors; EZ, ellipsoid zone of the photoreceptors; OS, outer-segments; RPE-IZ, the RPE-Bruch’s membrane (BM) complex and the RPE-photoreceptor interface (interdigitation zone, IZ). (**B** and **C**) Longitudinal OCT scans show changes in the outer retinal bands (scanning locations indicated as red lines in the B-AF images on top) during follow-up imaging of the left (**B**) and right (**C**) eyes. The RPE-IZ shows early attenuation with time and the EZ showed a wave-like pattern following the RPE-IZ attenuation, with hyperreflective deposits appearing above the RPE-IZ band beneath the waves at some locations (yellow arrows). The schematic B-AF images are the same as the 26th month B-AF image shown in the Figure 2E. (**D**) Hypothesis for the early progression of Best disease. The RPE-photoreceptor interface is the first to be damaged, leading to cellular dysfunction. The accumulation of membrane discs beneath the OS and damaged cone mitochondria in the macula further exacerbates the damage. This results in the death of RPE and photoreceptor cells in the macula. Corresponding changes in OCT imaging include initial thinning of RPE-IZ, followed by distortion of EZ band, accumulation of deposits, and thinning of the ONL, progressing in a center-to-peripheral pattern in the macula.

Histopathological analysis revealed RPE interface disruption alongside the accumulation of mitochondrial-positive deposits and outer segment deposits. In OCT imaging, from the outer to inner retina, the hyperreflective RPE-IZ layer includes the RPE-BM complex and RPE-PR interface (interdigitation zone, IZ), and the hyperreflective ellipsoid zone (EZ) includes photoreceptor mitochondria (Figure 6A). To investigate how these microstructures evolve during disease progression, we retrospectively analyzed macular OCT images from follow-up visits.

In the Q327E mutant left eye, the outer retinal layers initially appeared relatively intact at the first month, with a straight EZ and a defined RPE-IZ layer consisting of both the IZ and the RPE-BM complex hyporeflective zones (Figure 6B). By the seventh month, the RPE-IZ showed slight attenuation. At the 13th month, the IZ band had almost disappeared and the EZ was slightly attenuated. By the 26th month, the EZ band showed a wave-like pattern. Beneath the waves of EZ and above the RPE-BM complex, hyperreflective signals resembling type 1 deposits identified in histopathology were observed (Figure 6B).

In the mutant right eye at baseline, the RPE-IZ already showed compromised integrity, with incomplete hyperreflective signals from the IZ, while the EZ layer appeared straight (Figure 6C). By the 7th month, the integrity of the EZ layer began to decrease and showed a wave-like pattern. Hyperreflective signals, similar to type 1 deposits, were observed beneath the wavy EZ (Figure 6C).

In summary, the RPE-PR interface was the earliest structure to show disruption during disease progression. This disruption likely impaired the renewal of photoreceptor membrane discs, leading to mitochondrial damage and accumulation, particularly in cones. Over time, this resulted in selective degeneration and loss of the central macula, with thinning of the ONL. The accumulation of type 1 deposits beneath the outer segments and mitochondrial deposits above the EZ likely drives retinal detachment from the RPE. These processes ultimately create a subretinal cleft containing two distinct types of deposits, which may contribute to the formation of vitelliform lesions in the central macula (Figure 6D).

## Discussion

In this study, we present what is, to our knowledge, the first NHP model of inherited macular dystrophies, carrying a heterozygous deleterious *BEST1p.Q327E* variant, which has not been previously reported in humans. This naturally occurring mutant model successfully replicates key clinical and pathological features of early BVMD, including a subfoveal cleft with hyperautofluorescent deposits, reduced BEST1 protein in the RPE, disrupted RPE-photoreceptor outer segment interface, and macular photoreceptor loss. Notably, similar to human Best disease, our model demonstrates damage and dysfunction localized to the macula, with relatively normal peripheral retina and ERG response.

Although we did not identify the exact *BEST1p.Q327E* mutation in humans, our search revealed several human pathological mutations that mimic the effect of Q327E. Our structural modeling reveals that the 325–332 loop, where Q327 resides, modulates the mobility and stability of the A195 helix, which is crucial for the aperture conformation of the BEST1 ion channel. Similar disruptions in the stability of the A195 helix would be introduced by other previously reported pathogenic mutants of human Best’s disease, including A195V (21), M325H (22), and I201T (23). These human mutants have been validated to dampen the BEST1 ion channel function (37, 38). Thus, our findings suggest that the modulation of the A195 helix in primates and humans, therefore, represents a novel molecular mechanism governing BEST1 channel function. These findings support that the underlying molecular mechanism in our macaque model is most likely replicated in human Best disease patients.

An important gap in knowledge of Best disease has been the lack of insights into earlier stages of Best disease. Given the difficulty in obtaining early-stage human retinal samples, BVMD tissue samples available for research are typically from late-stage disease, where significant degeneration and scarring have already occurred (24, 34, 39, 40). Even with the advent of clinical OCT imaging, these studies have largely focused on patients with symptomatic disease or obvious clinical findings of vitelliform disease. It has been challenging to study earlier stages of disease and the underlying pathological mechanisms that trigger progression of this condition. In this regard, our early-stage BVMD NHP model provides potential unique and important insights. In addition to the disruption of the RPE-photoreceptor interface that is a hallmark of Best disease, our model suggests that both photoreceptor and RPE degeneration could be components of the disease at an earlier stage.

Additionally, our NHP model revealed foveal outer nuclear layer (ONL) thinning, a hallmark of cone-selective pathology. Foveal ONL thinning has been consistently observed in human BVMD across disease stages (26). Although cone functional impairments, such as color vision deficits and reduced visual acuity, commonly appear somewhat later in disease progression, subtle cone dysfunction can be detected early in patients with *BEST1* mutations (41–43). The clinical delay in symptom manifestation is likely due to cone redundancy in the macula and the limitations of diagnostic tools in detecting early dysfunction. Nevertheless, these findings suggest that cone-specific functional decline may be an intrinsic feature of *BEST1*-related retinopathies.

Interestingly, we observed an unexpected and heretofore unappreciated accumulation of damaged mitochondrial deposits also in a central-to-peripheral gradient in the macular center, coinciding with the subfoveal cleft enlargement and central-to-peripheral ONL thinning. This finding suggests that cone mitochondria may be particularly vulnerable in response to BEST1 dysfunction in the RPE, contributing to the selective macular lesion and dysfunction in the disease. The buildup of damaged cone mitochondria may contribute to retinal detachment and subfoveal cleft formation. Additionally, damaged cone mitochondria could also increase reactive oxygen species (ROS) production and metabolic burden, exacerbating damage to the RPE and photoreceptors in the macula. This feed-forward damage in the specialized cone-enriched structure of the primate macula may explain the spatial distribution of pathological damage associated with pan-RPE BEST1 dysfunction. These findings raise the question of whether cones are more reliant on RPE function for mitochondrial maintenance compared with rods. Disruptions in outer segment renewal by the RPE may overwhelm cone mitochondria with lipid processing demands, leading to mitochondrial damage due to excessive lipid accumulation. Alternatively, cones may require more robust mitochondrial quality control, given their higher metabolic demands and dependence on oxidative metabolism in the mitochondria (44, 45). In contrast, rods generate energy primarily through glycolysis and have a lower dependency on oxygen (46, 47). Emerging evidence suggests that cones expel damaged mitochondria extracellularly for clearance, possibly with the assistance of Müller cells (48). It would be of interest to investigate whether RPE cells participate in clearing damaged cone mitochondria and how this affects overall mitochondrial function in cones.

A limitation of our study is that our identification of lipid-rich abnormal mitochondria within cones is based on spatial distribution rather than direct cell-specific markers. Current immunostaining techniques lack the specificity to distinguish mitochondria of different photoreceptor subtypes. Future advancements in mitochondrial lineage tracing and high-resolution imaging technologies will be crucial for more definitive validation of their cellular origin. Although the *BEST1p.Q327E* variant is rare, the existence of large macaque colonies and expanding genomic screening efforts provides feasible opportunities to identify additional carriers (19). Alternatively, CRISPR-Cas9 technology now offers the means to precisely introduce mutations in *BEST1* to establish reproducible NHP models for mechanistic and therapeutic studies (49, 50).

Regardless of the interpretation, our study provides support for the importance of animal models of macular disease. The specialized structure and function of the macula is pivotal for the high resolution visual acuity in humans and NHP. In addition, the unique cellular and environmental milieu of the macula and fovea is well recognized to be critical for diseases, including macular dystrophies and age-related macular degeneration. The latter is a major cause of blindness in the elderly, in which blinding complications, including geographic atrophy and choroidal neovascularization, have a major predisposition for the macula, as opposed to peripheral retina. Indeed, Best macular dystrophy, while exhibiting a general, retina-wide dysfunction of the RPE (51), exhibits pathologic and functional changes almost exclusively in the macula. The structure and function of the macaque macula is very similar to humans, including OCT manifestations (13), thereby being very insightful for macular disorders. In addition, the genetic similarities of macaques to humans likely leads to more accurate modeling of disease in macaques as compared to other animal models. Our model demonstrates the specific impact of a gene mutation that affects protein function in all RPE cells, both macular and peripheral, while specifically impacting structure and function in the macula. This potentially provides a more valuable model for testing of potential therapies, including gene therapy, which can translate more readily into human clinical trials.

## Methods

### Sex as a biological variable.

Our study examined male and female animals and identified one male macaque who carried the mutation for the Best Disease. We included an age and gender-matched macaque as a control.

### Animals.

All crab-eating macaques (*Macaca fascicularis*) were from Huazhen Biotechnology Co. Ltd, Guangzhou, China. The animals were maintained at approximately 26°C and 40% to 70% room humidity on a 12-hour/12-hour light/dark cycle. The animals had no surgical history.

### Color fundus photography, fundus autofluorescence, and OCT.

The animals were sedated with an intramuscular injection of Zoletil 50 (VIRBAC S.A.) (4 mg/kg) and topical application of proparacaine HCl (Alcaine, 0.5%; Alcon Laboratories, Geneva, Switzerland). Pupils were dilated with 0.5% tropicamide. Color fundus photographs were obtained with a conventional flash fundus camera (TRC-NW8, Topcon Corporation).

Spectral domain OCT was performed with Heidelberg HRA-OCT (Spectralis; Heidelberg Engineering GmbH, Heidelberg, Germany) to acquire simultaneous imaging of the retinal morphology and fundus autofluorescence (FAF), according to the standard manufacturer’s protocol. Retinal fluorescence was excited by 488 nm pulsed blue laser light and detected with a barrier filter at 500 nm. Animals were monitored by a trained technician and a veterinarian at all times.

### Analysis of the OCT imaging.

Central foveal retinal thickness was measured using a linear cross-sectional B scan passing through the foveal center and optic cup, with each B scan averaged from 30 frames. Macular thickness was assessed using high-resolution horizontal spectral-domain OCT cross-sectional B scans of the macula, consisting of 25 lines spaced 245 μm apart. For peripapillary retinal nerve fiber layer thickness (RNFLT) measurements, a glaucoma protocol with a single circular B scan of 12° diameter was performed. The segmentation of different retinal layers was generated by the Heidelberg Eye Explorer software and manually adjusted by a trained ophthalmologist when needed. The thickness measurement of different retinal layers was done automatically by the Heidelberg Eye Explorer software.

### Electroretinography.

While sedated, animals were dilated and dark adapted for 30 minutes. ERG technology (Roland consult RETImap Model 520 ERG, Germany) was used to was used to perform a standard flash ERG according to the approved protocol of the International Society for Clinical Electrophysiology of Vision (ISCEV) (52). Active electrodes were placed into the eyelid, a reference electrode was placed at both sides under the skin, and the ground electrode was placed near the tail of the animal. Electrode impedance was acceptable with the difference less than 2 KΩ. Measurements were recorded and displayed using the manufacturer’s software.

### Whole-genome shotgun sequencing.

Whole-genome shotgun sequencing (WGS) was performed following the protocol provided by the manufacturer. Blood samples were collected from the *Macaca fascicularis* for genomic DNA extraction. A total amount of 1 μg of DNA per sample was used as input material for the DNA library preparations. The sequencing library was generated using CLEANNGS DNA kit and index codes were added to each sample. The clustering of the index-coded samples was performed on a cBot Cluster Generation System using Novaseq 5000/6000 S4 Reagent Kit (Illumina). After cluster generation, the DNA libraries were sequenced on Illumina NovaSeq 6000 platform and 150 bp paired-end reads were generated.

### Bioinformatics analysis.

Sequencing reads were compared with reference *Macaca fascicularis* 5.0 genome (macFas5) by using BWA software, and the results were converted into bam format and sorted by samtools software. Verita TreKKer was used to do identify SNP/ InDels. Enliven was performed to do annotation for SNP/InDels/CNV/SV. Enlivenand and ANNOVAR were performed to do annotation for VCF (Variant Call Format) obtained in the previous effort. dbSNP, 1000 Genome, and other related existing databases (e.g., ACMG) were applied to characterize the detected variants. Given to the significance of exonic variants, gene transcript annotation databases, such as Consensus CDS, RefSeq, Ensembl, and UCSC, were also included to determine amino acid alternation. The frequency of variants in genes associated with inherited macular dystrophies, including *BEST1*, *ABCA4*, *EFEMP1*, *ELOVL4*, *IMPG1*, *PROML1*, *PRPH2*, *RS1,* and *TIMP3* were calculated using R.

### Sanger sequencing.

Blood samples were collected from the *Macaca fascicularis* for genomic DNA extraction and Sanger sequencing using standard methods. Briefly, genomic DNA was extracted using FastPure Blood DNA Isolation Mini Kit V2 (DC111, Vazyme). The genomic DNA was amplified by PCR using the 2X Easy Taq PCR SuperMix (AS111, TransGen Biotech) using the following program, 96°C for 5 minutes, 35 cycles of 94°C for 30 seconds, 55°C for 45 seconds, 68°C for 45 seconds, and the final extension at 72°C for 2 minutes. The primer sets for the PCR of *Macaca fascicularis* *BEST1* gene were designed in the NCBI website using forward primer, GCACCCATCTCCCCATTTCA and reverse primer, GCCAGGTCCTAACCTTCCAC. Sanger sequencing was performed using the BigDye Direct kit.

### Plasmids, cell culture, and transfection.

The Human embryonic kidney 293T cells (HEK293T) and Adult Retinal Pigment Epithelial cell line-19 (ARPE-19) used in this study were obtained from the American Type Culture Collection (ATCC). HEK293T and ARPE-19 were cultured in DMEM-HG (Invitrogen) supplemented with 10% (vol/vol) FBS, 100 U/mL penicillin, and 0.1 mg/mL streptomycin.

The cDNA of *Macaca fascicularis BEST1* (*mfBEST1*) was subcloned into a pCMV plasmid using PCR amplification. The *mfBEST1* WT, Q327E, and A195V plasmids were generated using PCR-based site-directed mutagenesis. Additionally, *mfBEST1* WT and Q327E cDNA were subcloned into the pLenti-EF1 lentiviral vector, and lentivirus production was performed by OBiO Technology (Shanghai) Co., Ltd.

For transient transfection, plasmids were transfected into 293T cells using jetPRIME (101000046; Polyplus). The *mfBEST1* plasmids were transfected at a 9:1 ratio with pEGFP-N1 plasmid to show the transfection efficiency. Cells were collected after 48 hour transfection for electrophysiology or Western blot analysis.

To establish ARPE-19 cell lines stably expressing *mfBEST1* WT or Q327E, cells were infected at a multiplicity of infection (MOI) of 8. After 48 hours of incubation, puromycin selection (2 μg/mL) was applied to eliminate nontransfected cells, with selection maintained until complete cell death in the negative control group. Stable ARPE-19 cell lines were collected after two passages for Western blot analysis.

### Western blot.

For Western blot analysis, plasmid-transfected or virus-infected cells were lysed using RIPA buffer (Solarbio) supplemented with a protease inhibitor cocktail (P6730, Solarbio). Lysates were clarified by centrifugation, and protein concentration was determined using the BCA assay. Equal amounts of total protein were resolved on an 8% SDS-PAGE gel, transferred to a PVDF membrane, and blocked with 5% skimmed milk in TBST for 1 hour at room temperature. The membranes were then incubated overnight at 4°C with primary antibodies against BEST1 (Cat ab2182, Abcam) and ACTIN (Cat 4967, CST). After washing, membranes were incubated with HRP-conjugated secondary antibodies (Cat 7074S, CST) for 1 hour at room temperature. Immunoreactive bands were detected using a chemiluminescence detection kit (Thermo Fisher Scientific).

### Electrophysiology in cultured cells.

Anion channel activities were measured in HEK293T cells using the whole-cell patch-clamp techniques reported previously (10). Briefly, cells were transferred into a bath mounted on a stage with an inverted microscope (IX-70; Olympus, Tokyo, Japan). The voltage and current recordings were performed at room temperature (22–25°C). Patch pipettes with a free-tip resistance of approximately 2 to 5 MΩ were connected to the head stage of a patch-clamp amplifier (Axon muticlamp700B; Molecular Devices). pCLAMP software v. 10.2 and Digidata-1440A (Molecular Devices) were used to acquire data and apply command pulses. Silver chloride reference electrodes were connected to the bath via a 1.5% agar bridge containing 3 M KCl solution. Voltage and current traces were stored and analyzed using Clampfit v. 10.2 and Origin v. 8.0 (OriginLab Corp). Currents were sampled at 5 kHz. All data were low pass filtered at 1 kHz.

The bath solution for the whole-cell patch clamp contained 146 mM N-methyl-d-glucamine-Cl (NMGD-Cl), 1 mM CaCl_2_, 1 mM MgCl_2_, 5 mM glucose, and 10 mM HEPES (pH 7.4). The pipette solution contained 148 mM NMDG-Cl, 1 mM MgCl2, 3 mM MgATP, 10 mM HEPES, and 5 mM ethylene glycol tetraacetic acid (EGTA) (pH 7.2). The free Ca2+ concentrations of the buffer solutions were fixed to 1 μM by adjusting the Ca2+ chelator EGTA (5 mM) and CaCl_2_ concentrations using WEBMAX-C software (http://www.stanford.edu/-cpatton/maxc.html). To determine the current–voltage (I-V) relationship, the clamp mode was shifted to voltage clamp mode, and the I-V curve was obtained by applying step pulses from −100 to 100 mV (voltage interval: 20 mV; duration: 2 seconds; holding potential: 0 mV). We only choose the EGFP-positive cell to perform the whole-cell patch clamp.

### Tissue embedding and IHC.

At the study’s endpoint, the eyes were enucleated at necropsy. After removing the anterior segment by dissecting along the limbus to remove the cornea, lens, and vitreous body, the posterior eye cup was fixed with 4% paraformaldehyde overnight at 4°C then rinsed with phosphate-buffered saline (PBS). The tissue was cryoprotected with 30% sucrose in PBS overnight. The macular and peripheral regions of the posterior eye cup were then separated, embedded in optimal cutting temperature (OCT) medium, and cryosectioned at 15 μm using a cryostat (CM3050 S; Leica). For IHC, sections were washed with PBST, blocked with blocking buffer (5% BSA and 0.5% Triton X-100 in PBST) for one hours at room temperature, then incubated in primary antibody overnight at 4°C, followed by Alexa Fluor 488 (Cat A21206, Invitrogen) and 647-conjugated secondary antibodies (Cat A31571, Invitrogen) for 1 additional hour at room temperature. Primary antibodies against BEST1 (1:200, ab2182, Abcam), RPE65 (1:200, AB231782, Abcam), EZRIN (1:200, Ab4069, Abcam), and MCT1 (1:200, sc-365501, SantaCruz) were used. The BODIPY 493/503 dye (HY-W090090, MCE), WHEAT GERM AGGLUTININ (W11261, Thermo Fisher Scientific) were incubated similar to the secondary antibodies. The specific stain of pathology, including H&E and oil red were performed in Cryosections. Histological sections were imaged using a confocal microscope (LSM 980 Basic Operation, Carl Zeiss) and (TissueFAXS V6.0, TissueGnostics). Three-dimensional reconstruction was performed with Imaris 9.1.0 (Bitplane, Zürich, Switzerland).

### Analysis of RPE density.

RPE density analysis was performed at three specific retinal regions: fovea, parafovea/perifovea, and periphery using ImageJ software. Sections from the macular region embedding displaying visible foveal structures were categorized as fovea, while those without visible foveal structures were classified as parafovea/perifovea. Sections from the peripheral retina were considered the periphery. Each region of retina was represented by three-to-five nonadjacent sections, and the average of these measurements from each slide was calculated to ensure reliable data. The DAPI and RPE65 were used to label the nuclei and RPE cells.

### Statistics.

All quantitative data are presented as means ± SEM and analyzed by a 2-tailed unpaired Student’s *t* test (two groups) or 1-way ANOVA (>2 groups) with a Turkey’s post hoc comparisons test. *P* ≤ 0.05 was considered as statistically significant. The outliers were calculated and excluded by the ROUT method of GraphPad Prism.

### Study approval.

All animal procedures adhered to the ARVO Statement for the Use of Animals in Ophthalmic and Vision Research and were approved and monitored by the Institutional Animal Care and Use Committee of Zhongshan Ophthalmic Center (no. W2021023).

### Data availability.

There is no restriction on data availability. All data described in this report including supplemental materials are available in the report and the Supporting Data Values file.

## Author contributions

XL, EJD, and WY conceived and supervised the work. WY, MX, and XL designed research. WY, MX, and YX contributed to the investigation, data curation, writing of the original draft, and project administration. YC, ZY, and XM performed the ophthalmic examination. YZ performed the whole-cell patch. LZ, LS, XM, ZS, WQ, YL, AQ, KZ, and LO analyzed data. SC assisted in the protein structure analysis. WY, MX, YX, and XL wrote the manuscript. EJD, WY, and XL contributed to conceptualization, visualization, reviewing and editing, and funding acquisition.

## Supplementary Material

Supplemental data

Unedited blot and gel images

Supporting data values

## Version 1 05/06/2025

In-Press Preview

## Version 2 06/23/2025

Electronic publication

## Version 3 06/25/2025

Elia J. Duh's affiliation was corrected
