# Supplementary material for: A spontaneous nonhuman primate model of inherited retinal degeneration
Source: JCI Insight. 2025 May 6;10(12):e190807. doi: 10.1172/jci.insight.190807 (PMC12220943; doi:10.1172/jci.insight.190807)
Supplement: Supplemental data [file jciinsight-10-190807-s251.pdf]

Supplementary figures and legends

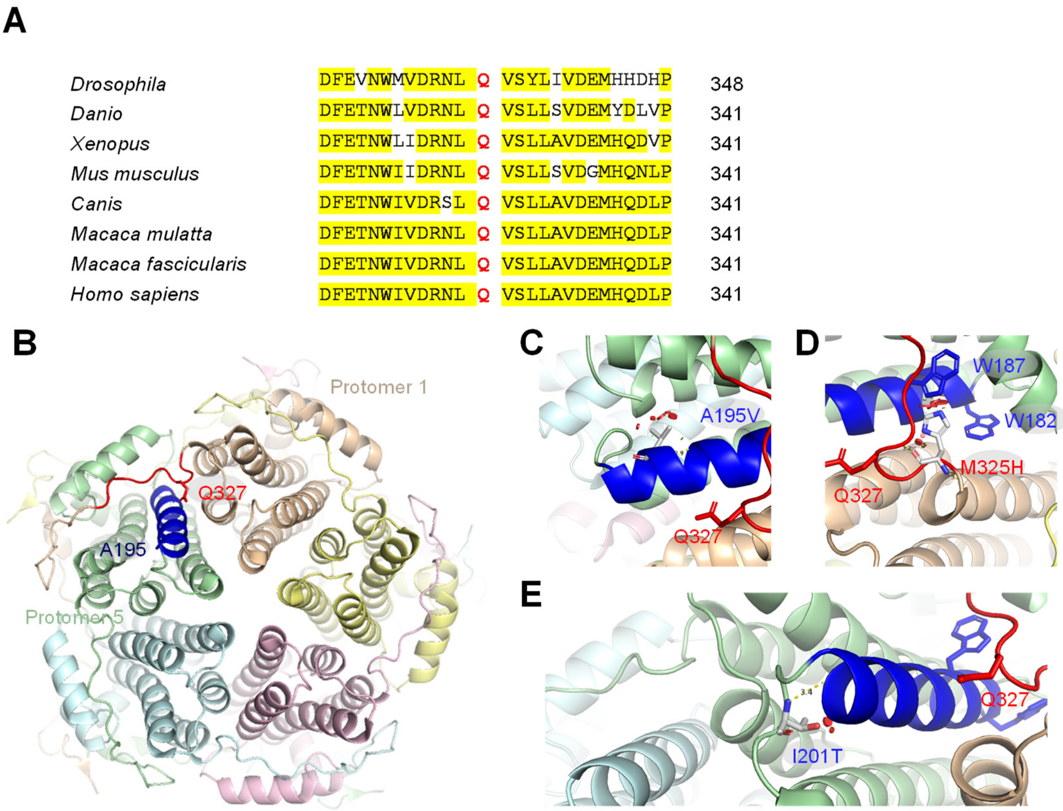

Figure S1. Pathogenic mutants of human Best's disease disrupt the stability of the A195 helix. **(A)** Protein sequence alignment demonstrates that the Q327 residue is highly conserved across vertebrate species. **(B)** Bottom view of the human Bestrophin-1 (hBest1) pentamer structure (PDB: 8D1K). The 325-332 loop (highlighted in red) of protomer 1 modulates the A195 helix (highlighted in blue) of the adjacent protomer. The side chains of residues Q327 and A195 are shown as sticks. **(C)** The A195V variant induces a disruption and destabilizes the A195 helix. **(D)** The M325H variant causes a destabilization of the interaction between residues W182 and W187 in the A195 helix. **(E)** The I201T variant impairs the mobility of the A195 helix.

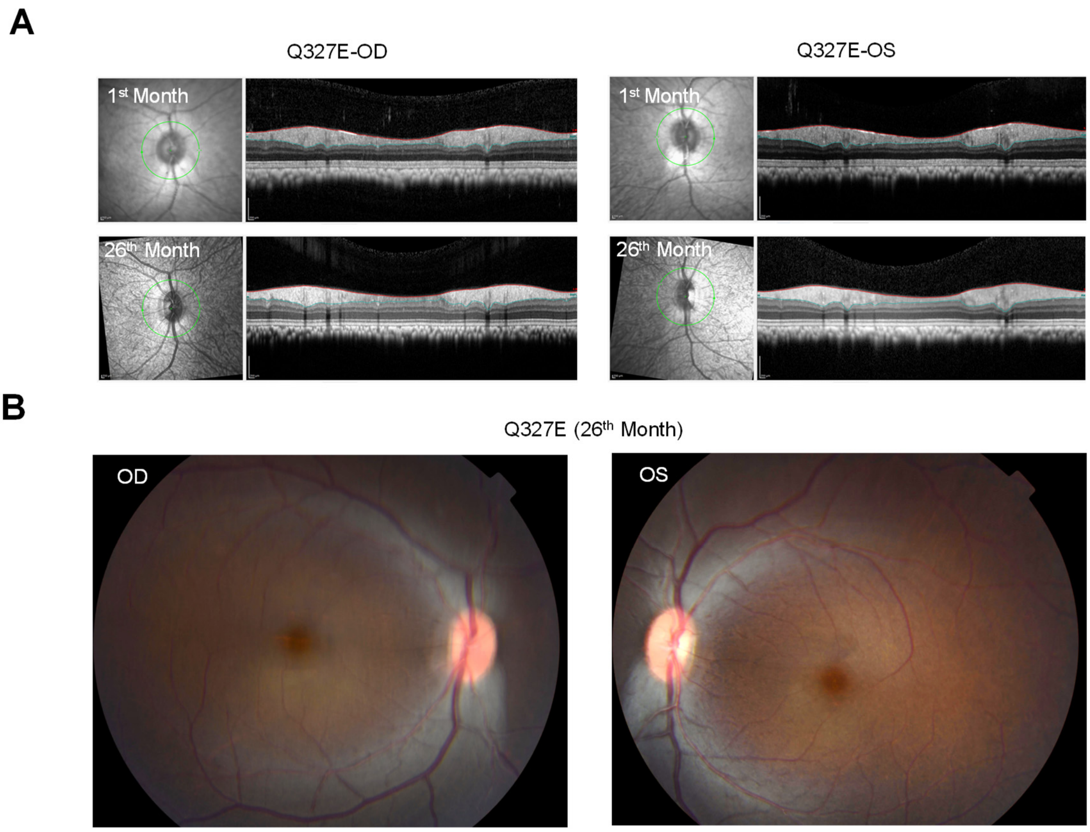

Figure S2. The inner retina thickness and fundus examination were normal in the mutant animal. (A) OCT scans of the peripapillary retinal nerve fiber layer (RNFL) from the first and the last visit of the mutant macaque. (B) Color fundus photography of the mutant animal showed no obvious pathologic finding at the last follow-up visit (9.8 years old).

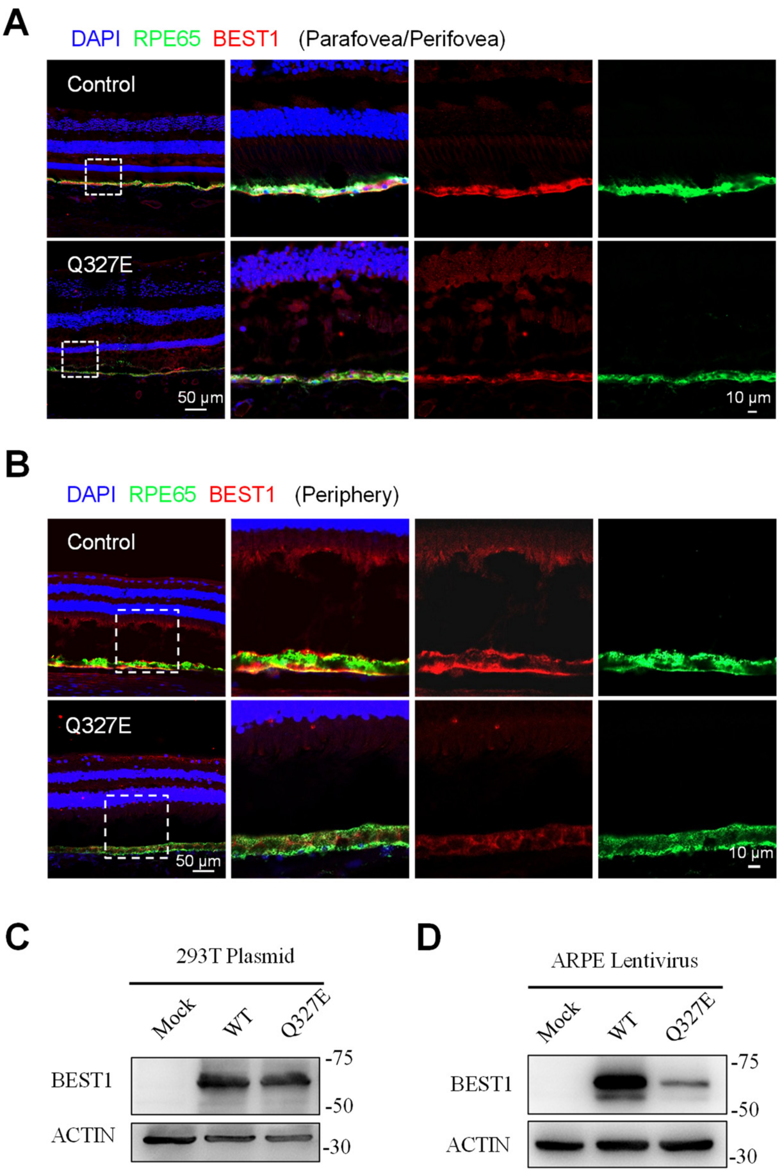

Figure S3. The Q327E mutation impare the BEST1 protein stability *in vivo* and *in vitro*. (A-B) Immunostaining for BEST1 protein (red) and RPE cell marker RPE65 (green), with nuclei stained with DAPI (blue). The BEST1 signal was largely reduced in both the parafoveal/perifoveal (A) and peripheral (B) retina of the mutant animal. **(C)** In 293T cells with transient expression of WT BEST1 and Q327E-mutant BEST1, no significant difference in protein levels was observed. **(D)** In ARPE-19 cells with stable lentivirus-mediated expression of WT BEST1 or Q327E-mutant BEST1, the Q327E mutant exhibited significantly lower protein levels compared to WT.

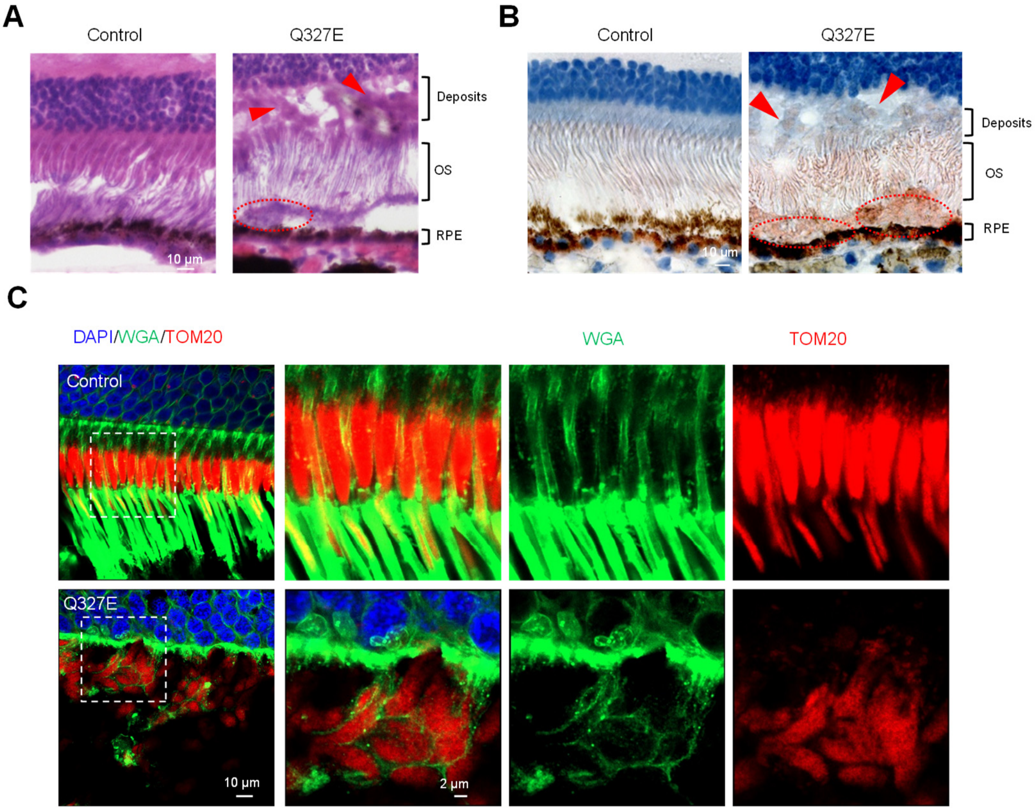

Figure S4. Histopathological changes in the mutant macaque. (A) Hematoxylin and eosin (H&E) staining revealed characteristic histopathological changes in the parafovea/perifovea of the mutant animal. Two types of deposits beneath (red arrowheads) and above (red circles) the disrupted outer-segment (OS) layer were observed. (B) Oil Red O staining showed similar lipid accumulation in the parafovea/perifovea of the mutant retina, indicating the presence of lipid deposits, as shown by the red arrowheads and above red circles. (C) Immunostaining for WGA (green) and TOM20 (red) revealed the internal location of the TOM20-positive deposit relative to the photoreceptor cell membranes. WGA, Wheat Germ Agglutinin.
