# Supplementary material for: A spontaneous nonhuman primate model of inherited retinal degeneration
Source: JCI Insight. 2025 May 6;10(12):e190807. doi: 10.1172/jci.insight.190807 (PMC12220943; doi:10.1172/jci.insight.190807)

Full unedited gel for Figure S3 C

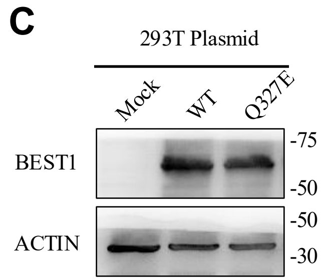

Figure S3 C in the manuscript

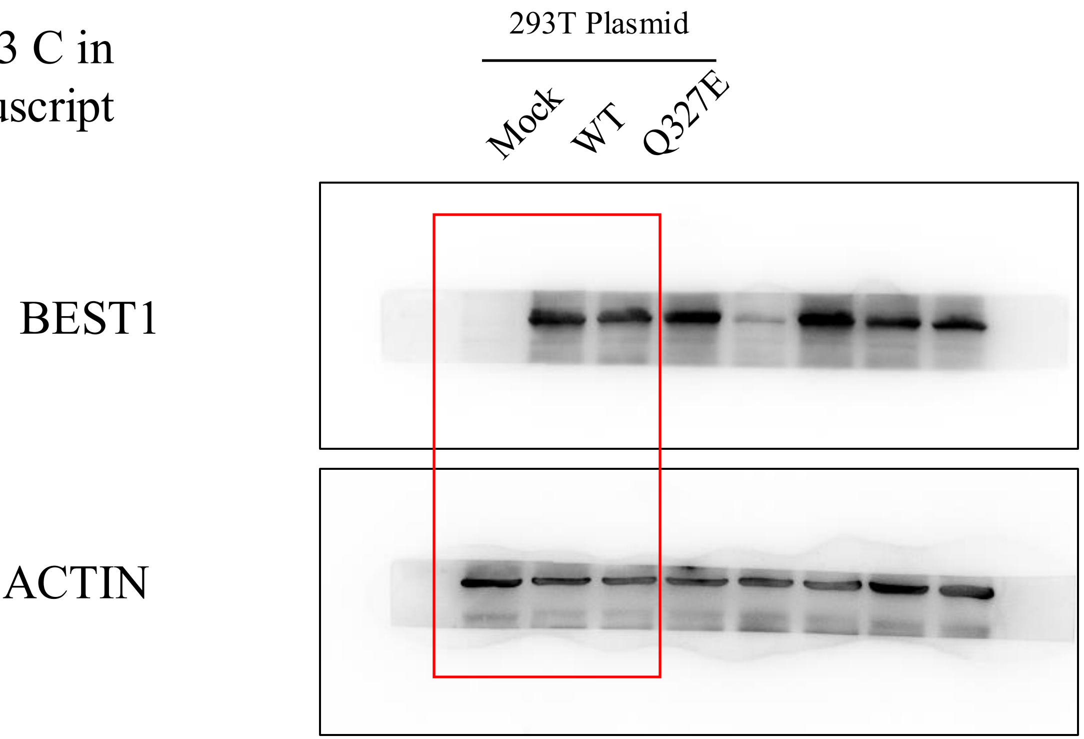

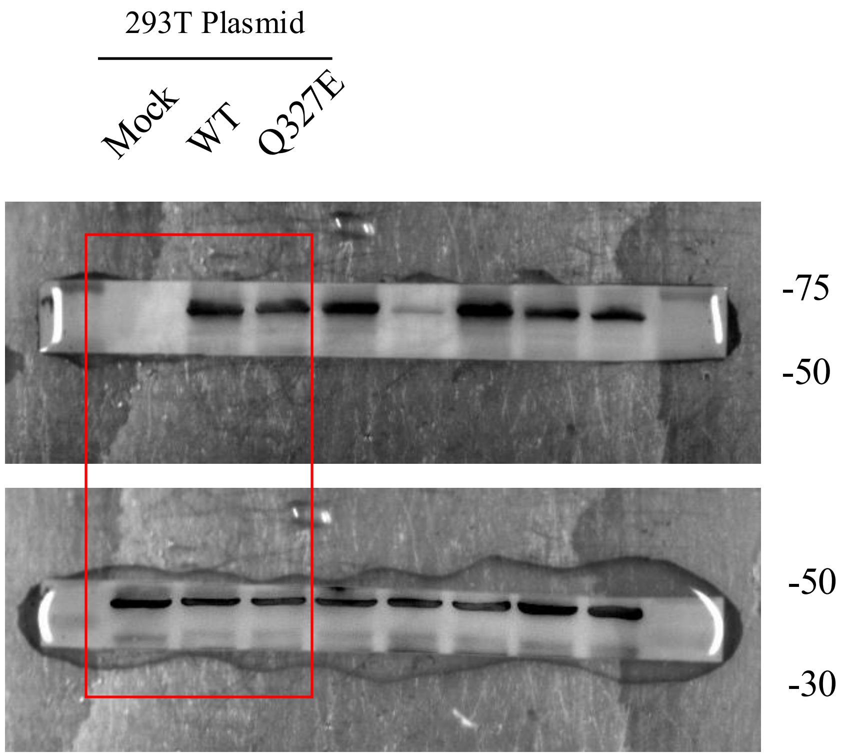

Full unedited gel for Figure S3 D

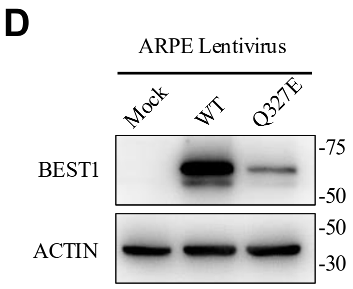

Figure S3 D in the manuscript

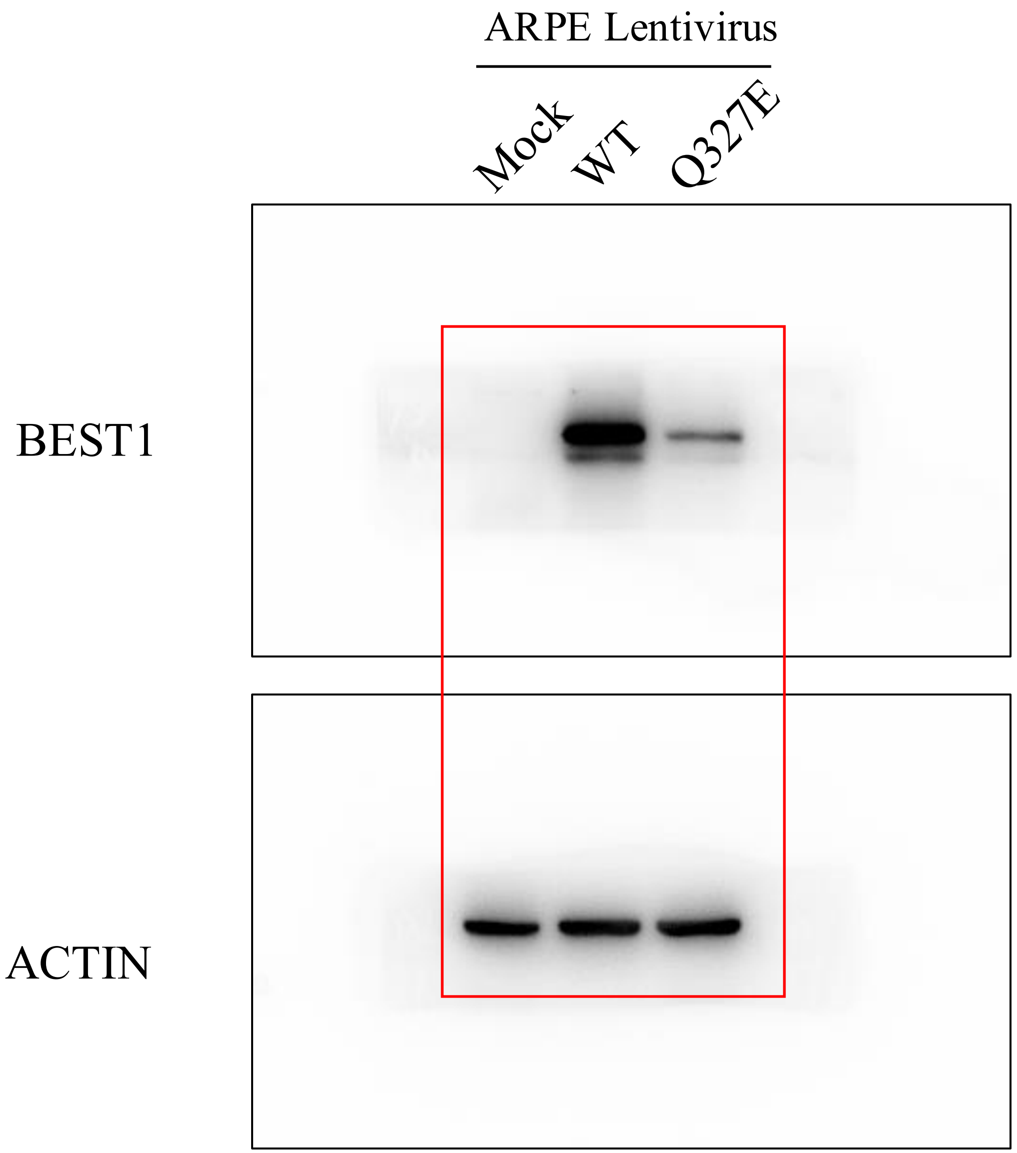

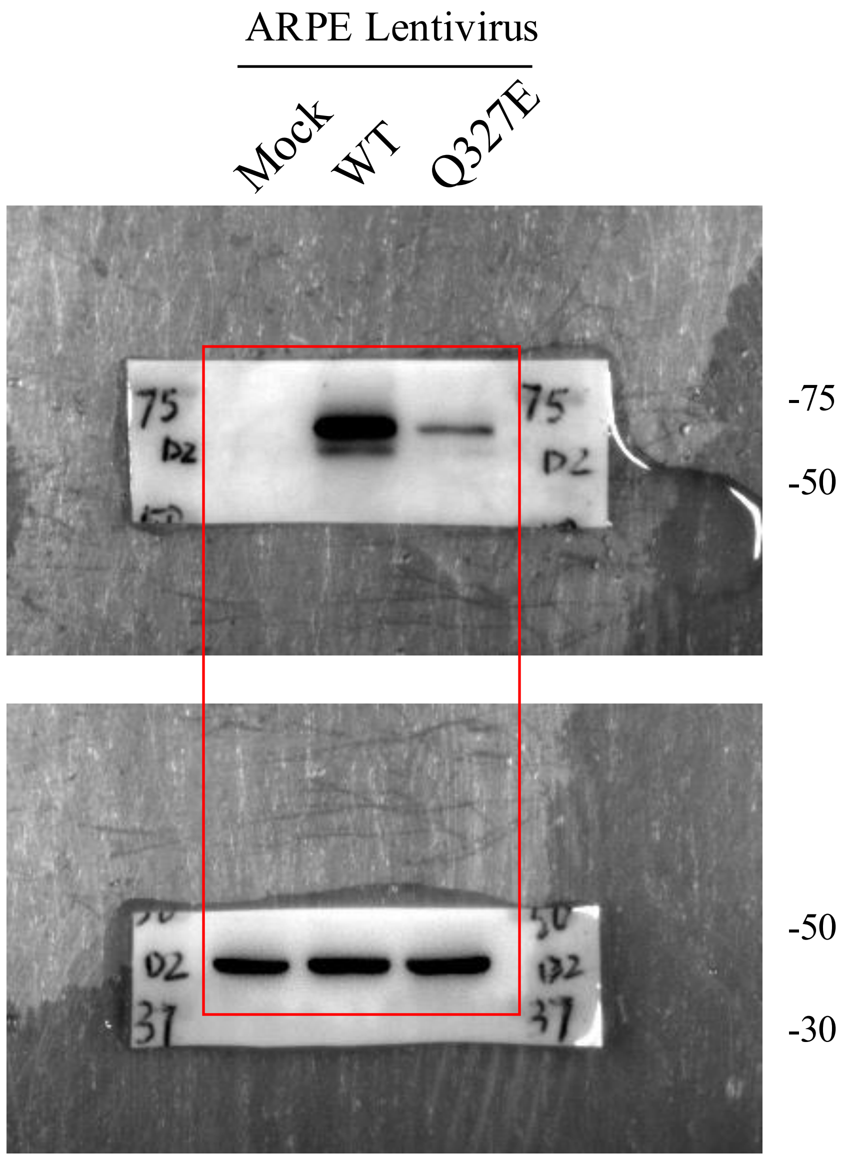

Supplement: Unedited blot and gel images [file jciinsight-10-190807-s252.pdf]
